# Factors Associated with the Quality-of-Life of Young Unpaid Carers: A Systematic Review of the Evidence from 2003 to 2019

**DOI:** 10.3390/ijerph20064807

**Published:** 2023-03-09

**Authors:** Camille Bou

**Affiliations:** NIHR School for Social Care Research, London School of Economics and Political Sciences, London WC2A 2AE, UK; c.l.bou@lse.ac.uk

**Keywords:** young carers, young adult carers, quality of life, systematic review

## Abstract

The aim of this review was to identify factors influencing the quality of life (QoL) of young people providing care for family members with chronic illnesses, disabilities, and/or mental health and substance abuse problems (young unpaid carers; YC), as well as the social-care related QoL measures. Focused and broad search strategies were performed in four databases, identifying 3145 articles. Following screening, lateral searches, and quality appraisal, 54 studies were included for synthesis. An inductive approach was used to synthesise the findings, grouping factors associated with YC QoL into interrelated themes: “*perceived normality of role and identifying as a carer*”, “*social support from formal and unpaid networks*”, “*caring demands and their impact*”, and “*coping strategies*”. No social-care related QoL measures for YC were found. This systematic review provides groundwork for the development of such a tool and emphasises the need for further studies allowing the investigation of the interrelated factors affecting YC QoL.

## 1. Introduction

Unpaid carers—individuals providing unpaid care to an older, ill, and/or disabled family member or partner—are a central group in health and social care policy. In England alone, there was an estimated 5.4 million people providing unpaid care in 2011 [1], with their number growing exponentially over the last couple of years. Although 50–64 year old women are most likely to undertake caring, children, adolescents, and young adults can also be carers [1]. The census estimated an overall 491,000 carers aged 24 or younger in the UK, including 166,363 in England [2]. The term *young unpaid carer* (YC) is used in this study to combine young carers and young adult carers together (i.e., individuals aged 24 and below providing unpaid care for family members who have chronic illnesses, disabilities, and/or mental health and substance abuse problems).

Unpaid carers require policy attention, as they can experience *caregiver burden*—the “*multiple physical, psychological, social, and financial stressors associated with caregiving*” [3]. There exists much evidence demonstrating caregiver burden to be inversely related with carers’ quality of life (QoL; e.g., [4]). Indeed, carers are more likely to have mental and physical health problems [5,6], feel socially isolated [7], and face financial difficulties [8] than noncarers. The impact of caregiver burden is particularly exacerbated if the pathway into caring is perceived as a necessity rather than a choice [9,10,11]. Yet, carers also report positive aspects of their roles, such as learning new skills, feelings of fulfilment, and a closer relationship with their care recipient [10]; these can moderate the relationship between caregiver burden and depression [9], and foster positive outcomes for the care recipient [12] (e.g., by exerting a protective effect on risk of institutionalisation [13]).

For YCs, caring takes place in tandem with key life stages and developmental milestones—from childhood, to adolescence, to young adulthood—leading the expectation that their experienced burden may differ from adult carers, due to the heightened instabilities associated with transitional periods of life. The United Kingdom (UK) was one of the first countries to recognise the needs of YCs as different from those of adult carers, and to include YC support policies in government strategies and legislative frameworks [14]. In England, the *Care Act 2014* promotes a better understanding of carer wellbeing. YCs are eligible for *Carer’s Assessments of Needs* (also offered to adult carers) and *Transition Assessments* (specific to YC who have already had their *Carer’s Assessment of Needs*, to be conducted before or upon their 18th birthday), to provide them and their families with information and support, helping them plan for their future and transition into adulthood [15]. Specifically, it states that YCs’ wellbeing must be central to the assessment, ensuring the creation of a person-centred plan accurately reflecting YCs’ individual needs and wishes, and the outcomes which matter to them [15]. The *Carer Action Plan 2018–2020* renewed the Government’s commitment to supporting carers, including specific actions for YC to identify “*the types of practical and emotional support that can enable […] positive transitions between the ages of 16–24*” [16]. The goals of these legislations, in many respects, reflect the World Health Organisation’s (WHO) definition of QoL: “*an individual’s perception of their position in life in the context of the culture and value systems in which they live and in relation to their goals, expectations, standards, and concerns*” [17]. Much like the *Care Act’s* person-centred approach to wellbeing, the WHO QoL carries a multitude of unique meanings, derived from an individual’s social and environmental context, physical and psychological state, and personal beliefs.

As exemplified by the UK context, YCs provide invaluable practical, personal, and economic support to family members; the protection of their wellbeing and QoL is of utmost importance. Awareness of YCs around the world is inconsistent, and although there exists research that investigated their experiences of care provision in different contexts [14], there is no systematic review collating the available evidence to explore how aspects of their caring experience may influence their QoL. Thus, this study aimed to complete a systematic review of the quantitative, qualitative, and mixed-method literature to identify factors influencing the QoL of YC as defined by the WHO, as well as tools available to measure YC social-care related QoL.

## 2. Materials and Methods

### 2.1. Protocol and Registration

This systematic review followed the methods outlined by the Preferred Reporting Items for Systematic Reviews and Meta-Analyses Protocols (PRISMA) statement [18]. The protocol is registered in the International Prospective Register of Systematic Reviews (PROSPERO)—CRD42019144592.

### 2.2. Information Sources and Search Strategy

The search strategy was inspired by the strategy of a similar systematic review [19]. Qualitative, quantitative, and mixed-method studies were searched using the electronic databases Medline Ovid, Embase Ovid, PsycINFO Ovid, and CINAHL Ebsco, adapting each strategy to the databases’ searching capabilities. “Snowballing” (i.e., checking the reference list of primary studies and reviews to find relevant papers) was additionally conducted to identify more potentially eligible studies [20].

Three concepts emerged from the study aim: (1) YC, (2) QoL, and (3) family members. The strategies were conducted twice on each database (Table 1): the focused search strategy (FSS) and the broad search strategy (BSS). The FSS only used terms defining YC (e.g., *young carer*). The BSS incorporated all three concepts whose terms were chosen to be as broad and sensitive as possible. For instance, the term caregiver* was limited by YC age groups, and the QoL concept included caregiver burden, attitudes, and willingness in line with the WHO definition. Conducting the search in this double manner ensured that all studies on YC were picked up, screened, and included according to the aims and inclusion criteria of this study.

### 2.3. Eligible Studies, and Inclusion and Exclusion Criteria

The target population included all individuals aged 24 and below, providing unpaid care to grandparents, parents, and siblings with chronic illnesses, disabilities, and/or mental health and/or substance abuse problems (based on the *young carer* and *young adult carer* definitions). Studies concerning caregivers of all ages were included if their samples consisted of 50% or more YCs out of their total sample size; if age-band sample sizes were not provided, the mean age of the sample was used to determine eligibility. If the sample was not defined according to these criteria, they were excluded. Studies were not excluded on the basis of the care recipients’ characteristics (e.g., illness type and severity, age, gender, and ethnicity), unless the care recipients were not parents, grandparents, or siblings of YC. Studies on paid carers, no matter their age, were excluded.

This review only included original articles published in English. There were no limits on the publication date, and the study context was limited to countries classified by GNI per capita as high-income as of August 2019 (when the literature search was completed). Papers which were not original articles (e.g., reviews, letters, commentaries, news, editorials, conference abstracts, validation, and protocols) were excluded. Doctoral theses were excluded. Interventional studies, including evaluations, were excluded as it was deemed the mechanism via which interventions impact QoL would complicate the interpretation of results through lack of clear inference between the intervention mechanism and the QoL outcome [19]. Quantitative studies were included if they used validated generic or disease-specific QoL measures with YC, and/or validated instruments (e.g., surveys) picking up factors influencing YC QoL. Qualitative studies were included if they conducted interviews and/or focus groups with YC to understand their experiences of providing care. Qualitative studies did not explicitly need to mention QoL but were required to demonstrate aspects of care provision influencing YC QoL, based on the WHO definition [17].

### 2.4. Study Selection

Electronic search results were downloaded into the Mendeley Reference Management Software, where titles and abstracts were screened to remove duplicates using a combined auto- and hand-searching approach [21], before screening the remaining title and abstracts against the predefined inclusion and exclusion criteria. Full articles were sought for all potentially relevant studies, within the limits of the access provided by the reviewer’s institutional library. Those not accessible through these means and irrelevant studies were removed. The protocol for resolving ambiguity concerning inclusion was through conversation with the study supervisor.

### 2.5. Risk of Bias and Quality Assessment

The Mixed Methods Appraisal Tool 2018 (MMAT) was used to assess the quality and risk of bias of the eligible studies [22]. The MMAT is divided into five appraisal sections: qualitative studies (Section 1), quantitative studies (Section 2, Section 3 and Section 4), and mixed-method studies (Section 5 along with Section 1, Section 2, Section 3 and Section 4). Study methods are assessed using the corresponding criteria and given an overall quality score ranging from 20% (one criterion met) to 100% (all criteria met). Eligible studies were scored, and ambiguity was resolved through conversation with the study supervisor. There currently exists no guidance on what constitutes an adequate quality threshold, except that excluding studies with low methodological quality is usually encouraged [22]. Thus, studies were excluded if they failed the MMAT screening questions and/or scored 20% or lower.

### 2.6. Data Extraction

Data from selected studies were extracted into Microsoft Excel to retrieve the following information: (1) author(s)/title, (2) year, (3) setting, (4) design, (5) sample (size, age, gender, relationship, and illness of care recipient), and (6) results. Quantitative outcomes included YC QoL measures, as well as univariate and/or bivariate results presenting factors influencing YC QoL. Qualitative outcomes included themes identified in the selected studies.

### 2.7. Synthesis

The study conducted a thematic analysis and a narrative synthesis method to identify, analyse, and report the results [23]. Themes were found inductively, driven by the findings of the eligible studies. Initially, independent themes were extracted from eligible qualitative studies using “vote counting” (i.e., calculating how many times different types of results across included studies appear; [23]). Quantitative outcomes were then extracted and grouped into independent themes, merging them with the themes initially identified from the qualitative studies if relevant.

## 3. Results

### 3.1. Study Selection

**Figure 1:**
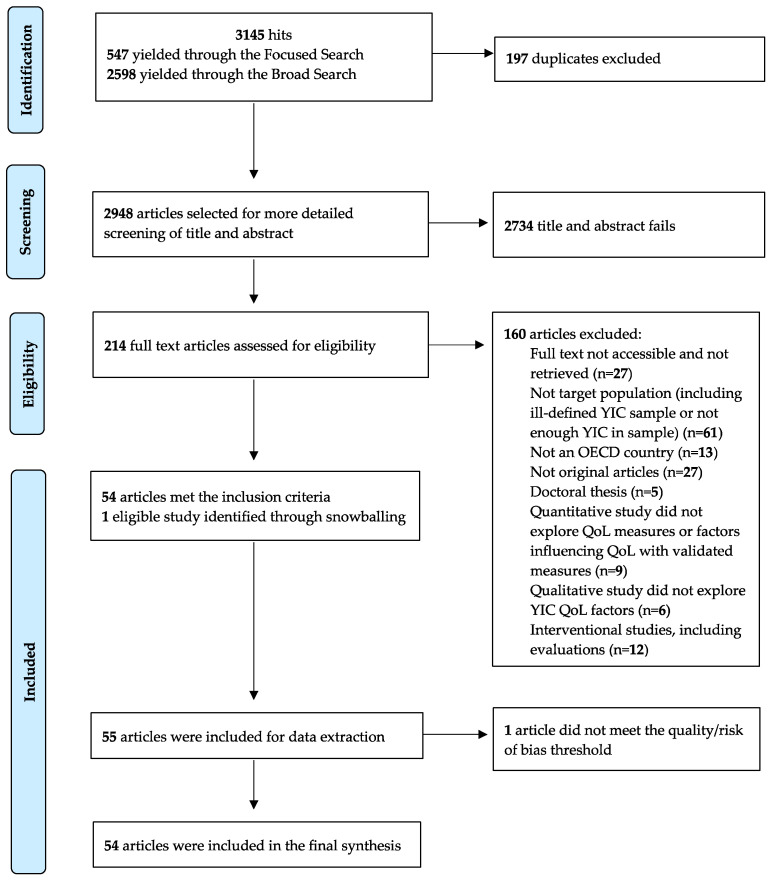
PRISMA flowchart of the selection process.

The search was conducted from May to August 2019. The FSS and BSS identified 3145 articles. Once duplicates were removed, 2948 articles remained. The titles and abstracts of these articles were screened, resulting in 214 potentially relevant articles. Following a thorough screening and selection process, 54 articles were included in the final synthesis. Figure 1 depicts the selection process.

### 3.2. Study and Participant Characteristics

Of the 54 studies included in the final synthesis, 27 were qualitative studies, 21 were quantitative studies, and six were mixed-method studies. One study was set in Israel, along with four set in Canada, 10 in the United States, 12 in Australia, 15 in the UK (with mentions of England, Scotland, Northern Ireland, and Wales), and the remaining 12 in other European countries including the Netherlands, Denmark, Norway, Germany, Switzerland, Sweden, and Belgium. Nineteen studies used *young carer* in their title to define their sample, with nine of those studies defining the age of a young carer as 18 and below. The term *young adult carer* was used in the title of eight studies to define their sample, with four studies defining the age of young adult carers as 16–24 years old.

The sample size of the included studies amounted to 11,699 YC, with 5770 females and 4747 males (n.b. some included studies had samples consisting of carers of different ages, with little detailed information as to the number of carers aged 24 and below in the sample. As such, the reported sample sizes are approximations based on the available sample size data of included studies). The care recipients in the studies were mostly parents (*n* = 42 out of 54 studies), followed by siblings (*n* = 19/55) and grandparents (*n* = 12/55). Care recipients’ chronic impairments varied, with reports of mental illness and substance abuse problems, Alzheimer’s disease and dementia, multiple sclerosis, Huntington’s disease, learning difficulties, autism, and Down syndrome.

Quantitative studies revealed that no YC social-care-related QoL measure currently exists. Instead, scales measuring the extent of the effect of factors on YC QoL were used, such as the Young Carers of Parents Inventory for caregiving experiences (YCOPI; *n* = 6), the Strengths and Difficulties Questionnaire for emotional and behavioural difficulties (SDQ; *n* = 5), and the Multidimensional Assessment of Caring Activities (MACA; *n* = 4) to assess YC caregiving activity. The KIDSCREEN was used in two studies to assess health-related QoL.

### 3.3. Quality Assessment

Results from the MMAT showed that 30 studies scored 100% (22 of which were qualitative studies), 17 studies scored 80%, and seven studies scored 60%; one study scored 20% or lower and, was, therefore excluded. However, the MMAT score reflects the review author’s reporting of the eligible studies’ methods; as such, that is not to say that the excluded study is of poor quality. As intervention studies were excluded from the review, sections 2/3 of the MMAT (“quantitative randomised controlled trials” and “quantitative nonrandomised studies”, respectfully) were not included in the table.

### 3.4. Narrative Synthesis

The Supplementary Materials present the included studies with their MMAT scores, associated themes, and measures.

### 3.5. Perceived Normality of Role and Identifying as a Carer

The perceptions of YC about their role and the internalisation of their caring identity were common themes. They described their pathways into caring as sudden, gradual, or normal. “Sudden” YCs found themselves in the role following significant changes in family structure (e.g., illness of a single parent or birth of a disabled sibling) [24]. “Gradual” YCs took on the roles as they got older [24] or as the health of their care recipient worsened [25]; this made pinpointing the beginning of their care provision difficult [24,26]. Age influenced whether a YC was able to pinpoint a start to their caring role, with YCs of younger ages finding it more difficult [26].

“Normal” YCs described their pathway into caring as a natural process within the family life which was not always a conscious choice; once more, age influenced the level of awareness of YCs to their newfound role in the family structure, with adolescents being more aware of how their caring responsibilities changed their lives [24]. The nature of caring tasks also seemed to play a role, with tasks related to personal or intimate care [27] or medical care (e.g., giving injections), raising awareness of the exceptionality of YCs’ roles [28]. Nonetheless, many “normal” YCs did not consider their responsibilities to be exceptional, but rather “routine” and “everyday”, feeling like they were “helping” rather than “caring” [27]. Some YC reported being “used to” providing unpaid care and could not recall a time during which they were not caring [29]. Some considered it like a job over which they had no choice [30], and others viewed their caring as an expression of love and loyalty for the care recipient [31]. In parallel, the term *parentification* was often used in the literature to describe the role reversal between YC and their care recipients who were parents [25,32,33,34,35]. Indeed, some YCs felt they “did not really have a chance to be a kid” [25] and struggled to find a balance between being a child/teenager and a carer [36,37], but “feeling like a parent” was also used by YCs to denote their added responsibilities rather than actual role reversal [35]. YCs caring for their grandparents reported similar feelings of role reversal or normative exchange [38]. YCs of siblings also placed boundaries on the tasks they performed if they were not the primary carers to prevent the blurring of their different familial roles [37,39].

The internalisation of young people’s caring identity was estimated to be influenced in part by the level of awareness and knowledge around YCs [24], as proven by some YCs gaining awareness of their role after meeting other YCs through support services or comparing themselves with noncarer peers [27,29]. However, reluctance to call oneself a YC was also motivated by YCs yearning for normalcy. Rather than labelling the exchange of assistance as imbalanced through the carer label, which often was associated with guilt, YCs of parents took pride in reciprocating care and supporting their families, claiming it was for their “own benefit” [40]. The young people who did identify as YCs reported differences in their willingness to disclose their identity [41]. Reasons for this varied; beyond the reasons related to accepting the change of their role within the family structure that were mentioned in the previous paragraph, YCs also stated keeping their family situation private (including the privacy of their care recipient) [41], fear of family separation by social services, and fear of stigma and social judgement associated with the care recipient’s illness as reasons to not disclose their caring identities [27,30,37,42,43,44].

### 3.6. Social Support from Formal and Unpaid Networks

YCs seemed to recognise that identifying as a YC could help them gain recognition for their roles and obtain the support they need and deserve [27]. Social support from formal and unpaid networks was another prominent theme which stood out from the literature. Findings suggest that when YCs did seek support, they better adjusted to caring [45,46]. However, the social capital of YCs varied, with some YCs better supported by formal and unpaid networks than others [47]. Generally, the social network of YCs consisted of friends, family members, school staff including teachers, health and social care professionals, and support workers.

YCs valued support from understanding noncarer friends [36,37,41,48,49] and other YCs with whom they could exchange coping methods [24,48,50,51]; however, for some YCs, caring took up much of their time and attention and, as such, hindered the development of friendships through sacrificed social opportunities [26,35,47,48,51,52], or not feeling “like their age” with regard to their noncarer peers [51]. In some cases, caring threatened the sustainability of YCs’ friendships, and they relied heavily on social media for their social interaction [37]. As a result of their caring, YCs reported feeling isolated [30,37,48,53], and care recipients felt guilty for limiting YCs’ opportunities to socialise. Feelings of isolation contributed to poor prosocial behaviour and a difficult transition to adulthood [54,55,56]. Self-stigma also played a role in peer relationship formation; YCs were often weary of exchanging confidences about their circumstances due to privacy concerns or fear of illness-related stigma [24,30]; self-stigma and isolation was compounded by low-income status [27,35]. When YCs did engage in social activities, they often felt guilt for leaving the care recipient alone and worried about them while they were away [44,47].

YCs also valued support from family members [36,49]. When they felt supported, appreciated, and acknowledged by their family members including their care recipient, they reported that caring improved their family closeness and their relationship with their care recipient [43,50,57]; however, if they felt taken for granted by their family, they viewed their caring negatively [50]. However, some YCs reported not wanting to share their burdens with their care recipient so as not to burden them [28]. Some YCs felt that updating their external family members was tiring and time-consuming, and that they often felt forgotten as the focus was on their care recipient; as such, YCs preferred to care alone than ask for help [24]. For young adult carers, moving out of the family home was a decision which produced a lot of guilt and worry over who would care in their place [26]; as a result, they often made decisions about their future with their care recipient in mind, e.g., choosing a postsecondary institution which was close enough to their home so they could continue caring [26,28,37].

With regard to health and social care services, YCs reported gaps in support provision, unmet needs, and barriers to access [26,43,51,58]. Barriers to access included service design issues, which influenced families’ trust that services could help, services’ lack of awareness of YC as a high-risk group, proximity, affordability, and availability of transport to physically access services, poor promotion of the services leading to lack of awareness of available support, and reluctance of families to connect with services by fear of intervention, scrutiny, or judgment by the system [24,26,43,49,53]. Communication with healthcare professionals was perceived as difficult for YCs; they could not always get information about their care recipients’ health due to patient confidentiality [24], and they expressed the need for empathetic, responsive professionals [51,55] who did not solely focus on their care recipients [35,59], as well as guidelines to help them better care for their care recipients [36,60]. YCs and their families accessed services such as respite, in-home occupational therapy or rehabilitation support, and community services, which relieved them of their care responsibilities; however, the quality of these services varied, and supply of these services was limited [35,43]. In combination with in-person support, web support, counselling, and group counselling were identified by YCs as potentially helpful [49].

Lastly, support from schools and teachers was frequently mentioned as an important part of YCs’ social capital [47]. While YCs in general seemed to enjoy going to school as it provided them with a break from caring and the possibility to socialise [47,61], YCs faced bullying and harassment at school [43,51], and did not feel protected by schools against this, with some YCs being home-schooled as a result [27]. YCs did not always share what was happening at home with their teachers, but sometimes wished they did as they felt it would help them better balance school with caring [24]. Conversely, some YCs wanted to keep their caring roles private to maintain separation between their homes and school, or to prevent preferential treatment from teachers [35,47]. YCs’ relationships with teachers varied, with some feeling supported and accommodated [47], and others perceiving teachers as insensitive to the realities of their caring situations and the impact on their studies [35,51]. They suggested that schools should support YCs through systems of pastoral care, as teachers were in the best positions to identify YCs, with primary school highlighted as an optimum time for support [59]. In general, evidence suggested that, when YCs did confide in teachers, their academic performance and enjoyment of education improved [47].

### 3.7. Caring Demands and Their Impact

Caring demands and their impact on YCs’ physical and mental health were prominent themes in the literature. Generally, YCs reported worse mental and physical health than their noncarer peers [62,63], as well as higher numbers of adverse childhood experiences [64]. Caring demands were directly correlated with stress and inversely correlated with life satisfaction [62,65,66,67]. Mental health problems included anxiety, depression [26,28,52,54,68,69,70], self-harm, substance use, and suicidal ideation [61]. Low self-esteem [67,68], feelings of guilt (e.g., of not doing enough to help), and worry about the care recipient were frequently mentioned by YC and negatively influenced their mental health [26,28,52,61], on top of general worries about their family’s finances, as well as preoccupations such as their schoolwork, their ability to form friendships, and whether they would experience bullying [61]. Indeed, further research suggests that YCs experienced bullying more often than noncarer peers [68], and that YCs felt the financial constraints of their families [43,53,55]. YCs reported feeling overwhelmed and exhausted [53], with difficulties sleeping and eating [26,61]. Age and gender were associated with YCs’ reporting of problems and worries in relation to their wellbeing, with older YCs more likely to convey both, and female YCs more likely to convey both [61,71]. Physical health problems were also noted, especially for older YCs [26]. The health impacts of caring demands expanded to other areas of YC lives, heavily affecting their education, as seen through some reports of absenteeism, lower grades, and diminished future aspirations [31,59,71,72,73,74]. Inability to pursue employment or to perform at work was also mentioned by YCs [26,37,41,43,71].

The nature and number of caring demands were dependent on the health of the care recipient (with poorer health related to increased caring demands), and whether the care recipient was a parent, which influenced whether the YC was a primary or secondary carer (with secondary YCs have less caring demands than primary YCs, and YCs caring for a parent having poorer psychosocial adjustment) [34,53,75,76]. Caring demands were categorised as instrumental (i.e., related to activities of daily living or doing administrative tasks and organising services) or emotional (e.g., keeping the care recipient company) tasks [33,50,58,69,70]. Emotional caring tasks and care tasks involving personal care of the care recipient were perceived as more burdensome than instrumental caring tasks involving household chores [33,50], and YCs caring for siblings with mental health problems reported that increased caring demands negatively influenced their relationship with them [66]. Similarly, high caring demands for parents seemed to negatively affect YC–parent relationships [69]. Caring demands were widely influenced by age gender, with females tending to have higher caring demands than males [34,61,66,74,76] and older YCs having more responsibilities [76].

### 3.8. Coping Strategies

YCs also described developing coping strategies to help them navigate their caring demands. One such strategy was benefit finding: reflecting on the gains of being a YC, such as family closeness, maturation, perspective on what is important, and relevant work experience for those in vocational caring fields [28,37,41,53,70,77,78] Benefit finding was inversely related to YC burden, mitigating negative health outcomes [65]; nonetheless, it was a weak predictor of adjustment, suggesting an unsustainability of the sole reliance of YCs on them [46,63]. Other coping mechanisms included seeking support, praying, solitude, and self-care [41,58]. Emotion-coping mechanisms were found to be more effective than problem-focused coping mechanisms, in line with the emotional impact of caring [25,33,35]. Internalising negative feelings such as guilt, shame, and worry was associated with maladaptive coping mechanisms [29,34,37,72]. Gratitude from family, the act of caring itself, and gaining information about the care-recipient’s illness were also perceived coping mechanisms [29,31,32,44,76], with the latter enabling YCs to improve their caring skills and caregiving confidence, the latter of which was associated with better adjustment [45]. However, overall social support was found to be the strongest predictor of YC adjustment, in opposition to coping and choice in caregiving which were weaker predictors [79].

## 4. Discussion

### 4.1. Summary of the Results and Implications

This systematic review aimed to synthesise the findings of primary research to identify factors influencing YC QoL. A better understanding of these factors could progress the support offered to YCs, as well as guide the formulation, implementation, and evaluation of interventions and policies aimed at improving their QoL.

The themes described in the narrative synthesis have undeniable associations and overlaps. The first theme, “*perceived normality of role and identifying as a carer*”, showed that young people often adopt caring seamlessly, making it difficult for them to pinpoint a start to their roles and recognise their exceptionality. Not identifying as a carer influences YCs’ perceptions of need for support and their ability to be enrolled and supported by services. Not all YCs want to be labelled as such, but they may still need support even if they do not realise it; thus, knowledge of the signs that a young person might be caring is crucial to signposting YCs to services.

As seen from the theme “*social support from formal and unpaid networks*”, YCs are prone to isolation from peers and services due to their caring demands. Many of YCs’ life decisions are not taken independently, but with the care recipient in mind. Therefore, a whole-family approach to the provision of support can help YCs adjust to their caring demands and help them plan for their futures in consideration of the specific contexts of their caring situations. Support offered by local charities such as YC peer groups stood out as particularly successful in the literature; however, these organisations are not able to—nor should they need to—replace or fill all the gaps in health and social care services. Barriers to access were identified in this review that need to be addressed to improve the support offered to YCs and their families, such as proximity and affordability of services, tailoring of services to fit families’ specific needs, promotion of services, and trust-building between families and services, including building awareness in health and social care professionals about YCs and the challenges they may face. Given their position in the family structure, YCs need to be given the choice to be included in the conversations about their care recipients’ health and wellbeing. The education system is another key player in the provision of YC support given that they are more likely to spot the signs of caring in youth and identify YC; moreover, teachers have been mentioned by YCs as the point of entry into accessing a wider range of health and social care services through identification. Awareness-building and information-sharing efforts can empower teachers with the tools to sensitively approach young people about their caring roles so they may seek support.

Supporting YCs is of utmost importance, as their “*caring demands*” have substantial and potentially long-standing impacts on their mental and physical health. This, in turn, can affect other areas important to YC QoL, such as the pursuit of education. The nature and number of caring demands is an important consideration, with emotional tasks having a bigger impact on mental health than instrumental tasks. Demographic factors such as gender and age were also found to influence caring demands, with females having higher caring demands, and YCs seemingly taking on more caring tasks as they got older. The “*coping with role*” theme suggests that YCs can build coping mechanisms and resiliency, helping them adjust to their added responsibilities, but relying on these strategies to improve YC QoL is unsustainable [46,63]. The systems that YC engage with—charities, health and social care services, and schools—have a duty to alleviate caring demands, offering YCs some respite, which can allow them to seek out opportunities which contribute positively to their QoL. Furthermore, the findings suggest that YCs with high caring demands are likely to be in families with financial constraints and, as such, rely on publicly funded services. Government-funding decisions impacting these services are likely to negatively affect YC and their families by limiting the amount of support they can access.

The heterogeneity of the studies included in this review made comparisons challenging. Similar methodologies (qualitative, quantitative, and mixed-method) were used but sample sizes, information collected on sample characteristics, and age groupings varied widely, which complicated the collation of demographic data and the association of demographic data to QoL. Sample selection in the included studies did not always resort to random sampling (especially qualitative studies which often made use of purposive sampling and relied on gatekeepers for recruitment) and, as such, may not reflect statistical representativeness. These are often methodological compromises which must be made when studying hard-to-reach populations such as YCs. Sociodemographic factors influence caring contexts; as such, they must be included in the design and analysis of future research including interventions—a systematic review of carer interventions unfortunately found that this was not always the case [80].

Lastly, the review findings revealed that no measure of YC social-care-related QoL currently exists. Instead, validated tools measure aspects of the YC experience which are likely to affect their QoL, e.g., the Multidimensional Assessment of Caring Activities (MACA) used in conjunction with the Positive and Negative Outcomes of Caring (PANOC) to assess YC caregiver burden. The KIDSCREEN shows promise in use in this population [81]; however, it is focused on evaluating health-related QoL. There is a clear gap in the literature and in our ability to evaluate social care services tailored to YC, which needs to be addressed. One way to do develop such a tool could be by psychometrically testing social-care-related QoL tools which are already available for adult carers, but have not been validated with YC samples. These measures include the Adult Social Care Outcome Toolkit (ASCOT), the Carer Experience Scale (CES), the CarerQoL, and the Adult Carer Quality of Life scale [82,83,84]. Mapping the themes found in this review and the domains of the scales extracted (Table 2), the ASCOT-Carer and Adult Carer-QoL seem promising given their themes seem to overlap with those found in this review.

### 4.2. Strengths and Limitations of This Review

The biggest strength of this review was its double search strategy. In addition to picking up potentially relevant studies, the FSS helped gain an understanding of how YC terms are used in the literature, and how many studies using these terms exist. Combined with the BSS, the review ensured that all potentially relevant studies were detected. This yielded a considerable number of hits, many of which were ineligible; albeit lengthy, the thorough screening and selection process and the limited number of articles found by snowballing gave the reviewer confidence that most eligible studies in the databases were identified.

The review methodology conversely contained limitations. It focused on high-income countries, to be applicable to a UK context. As such, the implications of the findings are unlikely to translate in low- and middle-income country contexts; it also only included studies in the English language. The review was conducted within a specific timeframe, which limited the publication year to August 2019. Further research is required to expand the search to include studies published beyond this date, refining the themes found in this review. Given that much of the health and social care literature from 2020–2022 focused on the impact of the COVID-19 pandemic, it is suggested that a separate study be conducted for publications from August 2019, to reflect the changes brought on by this unprecedented event at a system level, as well as its impact on YCs and their families. The systematic review steps were conducted by one reviewer, and it is usually encouraged that systematic reviews are conducted by at least two reviewers [22]. However, the eligibility and inclusion criteria of studies were made extensively clear for this purpose. Nevertheless, the MMAT’s critical appraisal score reflects the reviewer’s judgement based on their interpretation of eligible studies’ methods; thus, having a second reviewer for this stage could have provided additional insight. Lastly, the “vote-counting” method in thematic analysis is debated by researchers, as it equally weighs studies which may not be equal (e.g., sample sizes, significance, and effect sizes).

## 5. Conclusions

This systematic review revealed the complexity of YC lives, whose QoL is affected by interlinked and overlapping factors. A better understanding of factors influencing YC QoL could progress their identification and support, guiding the formulation, implementation, and evaluation of YC interventions and policies. However, differences between YC emerged in each theme, suggesting that a “one-size fits all” approach to support will not work; rather, support for YCs must be personalised and utilise a whole-family approach, taking into consideration the heterogeneity of caring situations. Further research is needed to empirically investigate the holistic influence of the themes of the review on YC QoL and develop a tool to evaluate their social-care related QoL. Similarly, research exploring these factors in more depth, as individual, primary outcomes, is needed. The YC evidence base is continuously growing, and a follow-on systematic literature review should be conducted to update the themes found in this study.

## Figures and Tables

**Table 1:** Example of focused and broad search strategy terms for Medline OVID.

Focused Search Strategy	Broad Search Strategy
Medline Ovid	
young carer*.mp **[53 hits]** young caregiver*.mp. **[24 hits]** young adult carer*.mp. **[4 hits]** young adult caregiver*.mp. **[4 hits]** caregiving youth.mp. **[4 hits]** 1 or 2 or 3 or 4 or 5 **[78 hits]** limit 6 to English language **[76 hits]**	Caregiver*/ **[33,376 hits]** limit 1 to (“preschool child (2 to 5 years)” or “child (6 to 12 years)” or “adolescent (13 to 18 years)” or “young adult (19 to 24 years)”) **[9474 hits]** “Child of impaired parents” **[5067 hits]** 2 or 3 [14,428 hits] grandparent.mp. or grandparents/ **[907 hits]** parents/or fathers/or mothers/or single parent/ **[103,820 hits]** Sibling*/or brother*/or sister*.mp. [mp=title, abstract, original title, name of substance word, subject heading word, floating sub-heading word, keyword heading word, organism supplementary concept word, protocol supplementary concept word, rare disease supplementary concept word, unique identifier, synonyms] **[43,349 hits]** 5 or 6 or 7 **[146,542 hits]** “Quality of life”/or qol/or ql/or hrqol/or hrql/or “health related quality of life”/or “health-related quality of life”/or life quality/or wellbeing/or life satisfaction/or “CarerQol”/or “SCRQol”/or “social care related quality of life”/ **[273,424 hits]** “cost of illness”/or caregiver burden.mp. **[27043 hits]** attitude/or motivation/or willingness/or perception/or need/ **[137,924 hits]** 9 or 10 or 11 **[634,680 hits]** 4 and 8 and 12 **[859 hits]** limit 18 to English language **[824 hits]**

**Table 2:** Comparison of social care QoL scale domains for adult carers with themes from the literature review representing factors affecting YC QoL. Boxes left blank indicate that no domain was found to match the YC outcome.

Scales and Their Domains/Systematic Review Themes	Perceived Normality of Role and Identifying as a Carer	Social Support from Formal and Unpaid Networks	Caring Demands and Their Impact	Coping Strategies
Carer-QoL	-	Support Relational problems	Social, mental, and physical health problems Financial problems	Fulfilment
ASCOT-Carer	Control over daily life	Feeling supported and encouraged	Occupation, personal safety	Space and time to be yourself
		Social participation		Self-care
CES	Control over caring	Support from family and friends	-	Activities outside of caring
		Assistance from organisations and the government		
		Getting on with the care-recipient		Fulfilment from caring
Adult Carer-QoL	Caring choice	Support for caring	Caring stress	Sense of value
			Ability to care	Personal growth
	Carer satisfaction		Money matters	Carer satisfaction

## Data Availability

The NIHR strongly supports the sharing of data in the most appropriate way, to help deliver research that maximises benefits to patients and the wider public, as well as the health and care system, which contributes to economic growth in the UK. In compliance with research integrity, this article has been made available to the scientific community with as few restrictions as feasible. The data used in this article are accessible through their respective DOIs.

## Supplementary Materials

The following supporting information can be downloaded at: https://www.mdpi.com/article/10.3390/ijerph20064807/s1, Table S1: Summary of study characteristics of identified studies, including MMAT score.
