# Supplementary material for: Factors Associated with the Quality-of-Life of Young Unpaid Carers: A Systematic Review of the Evidence from 2003 to 2019
Source: Int J Environ Res Public Health. 2023 Mar 9;20(6):4807. doi: 10.3390/ijerph20064807 (PMC10048985; doi:10.3390/ijerph20064807)
Supplement: Supplementary file 1 [file ijerph-20-04807-s001.zip › ijerph-2165421-supplementary.pdf]

Supplementary Table S1. Summary of study characteristics of identified studies, including MMAT score

Source	Year	Setting	Design (QT/QL/MM)	YIC Sample Size	Male/ Female	Age, Mean (SD) or range	Relationship to care-recipient	Illness of care-recipient	Theme/measures used	MMAT Score (%)
**Thomas, Stainton, Jackson et al.** 'Your friends don't understand': Invisibility and unmet need: In the lives of 'young carers'	2003	Wales, UK	QL (focus groups)	27	8/13	Range = 9-18<br>Mean=14	12 Parent (primary)<br>6 Parent (secondary)<br>5 Sibling (secondary)<br>4 Undisclosed	Parent<br>15 Sick/Disabled<br>3 Substance abuse/Mental illness<br><br>Sibling<br>3 Sick/Disabled<br>2 Behavioural problems	• Perceived normality of role and identifying as a carer (parentification; added responsibility rather than role reversal)<br>• Social Support from formal and unpaid networks (development of friendships; stigma and isolation compounded by low-income status; healthcare professionals and availability of social services; disclosure of identity to schools)<br>• Caring demands (emotional impact)<br>• Coping strategies (findings positives)	100%
**Cree.** Worries and problems of young carers: Issues for mental health	2003	Scotland, UK	MM (survey n=50 + interview n=11)	61	25/34	Range = 5-16	41% Parent<br>13% Sibling<br>46% Both	40% Physical disability<br>15% Problematic behaviour<br>13% Learning difficulty<br>10% mental illness	• Caring demands (general worries and problems as adolescents on top of those as a carer)<br>• Demographic factors: ethnicity (social support and caring demands), age (caring demands), gender (caring demands)<br>• Caring situation: length of time caring, existence of high/low risk group	100%

Supplementary Table S1. Summary of study characteristics of identified studies, including MMAT score

Source	Year	Setting	Design (QT/QL/MM)	YIC Sample Size	Male/ Female	Age, Mean (SD) or range	Relationship to care-recipient	Illness of care-recipient	Theme/measures used	MMAT Score (%)
**Eley.** 'If they don't recognize it, you've got to deal with it yourself': Gender, young caring and educational support	2004	Scotland, UK	QL (interview)	11	5/6	Range=10 -17<br>Mean=13	N/A	N/A	• Demographic factors: gender (caring expectations and demands)<br>• Perceived normality of role and identifying as a carer (including recognition from professionals)<br>• Social Support from formal and unpaid networks	100%
**Levine, Hunt, Halper et al.** Young adult caregivers: A first look at an unstudied population	2005	United States	QT (survey)	134	87.98/46.02	Range=18 -25	Female family member (65.8%/59.4%)<br>Grandmother (42.2%/24.1%)<br>Mother (7.4%/15.4%)	N/A	• Caring demands<br>• Coping strategies<br>• Social Support from formal and unpaid networks	80%
**Yahav, Vosburgh & Miller.** Emotional responses of children and adolescents to parents with multiple sclerosis.	2005	Israel	QT (survey)	56	32/24	Range=10 -18<br>Mean=14. 69 (2.2)	56 Parent<br>33 Mother<br>23 Father	Multiple Sclerosis	• Caring demands (gender effect)	60%
**Siskowski.** Young caregivers: effect of family health situations on	2006	United States	QT (survey)	6,714/ 11,029	~3343.57/~ 3370.43	Range=14 -15 (37.9%)	Parents	N/A	• Caring demands (gender and ethnicity effect)	80%

Supplementary Table S1. Summary of study characteristics of identified studies, including MMAT score

Source	Year	Setting	Design (QT/QL/MM)	YIC Sample Size	Male/ Female	Age, Mean (SD) or range	Relationship to care-recipient	Illness of care-recipient	Theme/measures used	MMAT Score (%)
school performance.						Range=16-18 (15.3%)				
**Moore & McArthur.** We're all in it together: Supporting young carers and their families in Australia	2007	Australia	QL (interview)	50	26/24	Range=9-24	26 Mother<br>16 Father/ Stepfather<br>19 Brother<br><br>14 cared for more than one relative	25 Alcohol/Substance abuse<br>18 Mental health<br>13 Physical disability<br>~17 Multiple conditions	• Perceived normality of role and identifying as a carer (disclosure of caring identity)<br>• Social Support from formal and unpaid networks (support from services, problems with services, reluctance to interact within family, promotion of services, impact of support)<br>• Caring demands (need for respite, financial hardships)	80%
**Bolas, Van Wersch & Flynn.** The well-being of young people who care for a dependent relative: an interpretative phenomenological analysis.	2007	England, UK	QL (interview)	5	3/2	Range=14-18<br>Mean=15.2	3 Mother<br>1 Step-father<br>1 Brother	N/A	• Perceived normality of role and identifying as a carer (making sense of identity and choice over caring; disclosure of caring identity)<br>• Social Support from formal and unpaid networks (isolation; illness-related stigma)	80%

Supplementary Table S1. Summary of study characteristics of identified studies, including MMAT score

Source	Year	Setting	Design (QT/QL/MM)	YIC Sample Size	Male/ Female	Age, Mean (SD) or range	Relationship to care-recipient	Illness of care-recipient	Theme/measures used	MMAT Score (%)
**Pakenham, Chiu, Bursnall et al.** Relations between social support, appraisal and coping and both positive and negative outcomes in young carers	2007	Australia	QT (survey)	100	28/72	Range=10-25<br>Mean = 16.28 (3.75)	Parent<br>76 Mother<br>16 Father<br>7 Both	68 Physical illness<br>18 Mental illness<br>7 Physical disability<br>2 Sensory disability<br>2 Brain injury<br>1 Alcohol and/or drug problem<br>2 Other<br>21 Parent has additional illness(es)/disability(s)	• Social Support from formal and unpaid networks (young people who gain support better adjust to caring)<br>• Caring demands (stress appraisal, distress) → Brief symptoms Inventory, Bradburn Affect Balance Scale, Satisfaction with Life Scale<br>• Coping strategies → Benefit Finding scale	80%
**Earley, Cushway, & Cassidy.** Children's perceptions and experiences of care giving: a focus group study	2007	Europe (exact setting not mentioned, supposed Ireland or UK based on academic affiliation)	QL (interview + 2 focus groups)	17	13/4	Range=10-16<br>FG1: Mean=13.11<br>FG2: Mean=11.39	4 Brother<br>4 Sister<br>5 Mother<br>1 Grandparent<br>4 more than one	7 Learning disability<br>3 Physical disability<br>5 Chronic Illness<br>2 Combination	• Perceived normality of role and identifying as a carer (perceptions of responsibility, identity and transition into adulthood)<br>• Social Support from formal and unpaid networks (social restrictions)<br>• Caring demands (stigma, bullying, burden of responsibility)<br>• Coping strategies (regulating, helping others)	100%
**McMahon & Luthar.** Defining Characteristics and Potential Consequences of	2007	United States	QT (survey)	356	163.76/192.24	Range=8-17<br>Mean=12.07	All Mothers	Mental health /Substance abuse	• Perceived normality of role and identifying as a carer (parentification)<br>• Caring demands → Child Caretaking Scale (caretaking	100%

Supplementary Table S1. Summary of study characteristics of identified studies, including MMAT score

Source	Year	Setting	Design (QT/QL/MM)	YIC Sample Size	Male/ Female	Age, Mean (SD) or range	Relationship to care-recipient	Illness of care-recipient	Theme/measures used	MMAT Score (%)
Caretaking Burden Among Children Living in Urban Poverty									burden from perspective of children), Behavioural Assessment System for Children (emotional-behavioural disturbance and competence in children), Clinical Maladjustment and School Maladjustment from the Self-Report of Personality (emotional distress and alienation from school), Internalizing Problems and Externalizing Problems from the Parent-Rating Scales (emotional-behavioural disturbance in the children from mother perspective)<br>• Demographic factors: ethnicity, SES	
**Fruhauf & Orel.** Developmental issues of grandchildren who provide care to grandparents	2008	United States	QL (interview)	34	15/19	17 people aged 7-17 Mean=12. 5,(3.12)<br><br>17 people aged 21-19 Mean=24. 12, (2.67)	All Grandparents, aged 67-93 years	N/A<br><br>(mentions of cognitive and/or physical limitations)	• Perceived normality of role and identifying as a carer (disclosure of caring identity)<br>• Social Support from formal and unpaid networks (school; family cohesion; worry and guilt)<br>• Caring demands (burden of responsibility, physical demands)	100%

Supplementary Table S1. Summary of study characteristics of identified studies, including MMAT score

Source	Year	Setting	Design (QT/QL/MM)	YIC Sample Size	Male/ Female	Age, Mean (SD) or range	Relationship to care-recipient	Illness of care-recipient	Theme/measures used	MMAT Score (%)
									• Coping strategies (regulating, respite)	
**Metzing-Blau & Schnepp.** Young carers in Germany: to live on as normal as possible - a grounded theory study.	2008	Germany	QL (interview)	41	16/25	Range = 8-19	23 Mothers (67.6%)<br>7 Fathers (20.6%)<br>1 Grandmother (2.9%)<br>2 Siblings (5.9%)<br>1 Parents and Grandparent (2.9%)	21 somatically ill: Multiple Sclerosis, Stroke, Parkinson's Disease, Asthma, Cardiac Insufficiency, Cancer<br>9 mental illness: Depression, Psychosis, PTSD<br>4 both (somatically as well as a mentally ill)	• Social Support from formal and unpaid networks (isolation; bureaucratic hurdles; distrust from support services)<br>• Demographic factors: SES	100%
**Östman.** Interviews with children of persons with a severe mental illness - Investigating their everyday situation	2008	Sweden	QL (interview)	8	3/5	Range=10-18	7 Mothers<br>1 Father	Mental Illness (schizophrenia and affective disorder)	• Perceived normality of role and identifying as a carer (disclosure of caring identity)<br>• Caring demands (stigma, feeling of loneliness, experience of fear and blame)<br>• Coping strategies (maturity, love for family)	100%
**Allen, Oyebode & Allen.** Having a father with young onset dementia: The impact on well-	2009	England, UK	QL (interview)	12	5/7	Range=13-23<br>Mean=19	All Fathers, aged 51–64 years	Young onset Dementia<br>Alzheimer's disease<br>Vascular dementia<br>Front-temporal dementia	• Perceived normality of role and identifying as a carer (reconfiguration of parental relationship; parentification; balancing responsibilities with youth)	100%

Supplementary Table S1. Summary of study characteristics of identified studies, including MMAT score

Source	Year	Setting	Design (QT/QL/MM)	YIC Sample Size	Male/ Female	Age, Mean (SD) or range	Relationship to care-recipient	Illness of care-recipient	Theme/measures used	MMAT Score (%)
being of young people									• Social Support from formal and unpaid networks (lack of help from relatives)<br>• Caring demands (physical and psychological strain, stigma, worry about parent, caregiving strain, difficulties related to illness)<br>• Coping strategies (problem or emotion focused)	
**Fraser & Pakenham.** Resilience in children of parents with mental illness: Relations between mental health literacy, social connectedness and coping, and both adjustment and caregiving	2009	Australia	QT (survey)	44	17/27	Range=12–17<br>Mean=13 (1.58)	33 Mother<br>4 Father<br>7 Both	19 Depression<br>11 BPD<br>14 Psychosis/Schizophrenia<br>8 Drug Abuse<br>4 Personality disorder<br>1 Eating disorder<br>7 Anxiety disorder	• Social Support from formal and unpaid networks (Social Connectedness Scale; isolation and prosocial behaviour)<br>• Caring demands (Children's Depression Inventory; Satisfaction with Life Scale; Strengths and Difficulties Questionnaire; Young Caregiver of Parents Inventory)<br>• Coping strategies (Responses to Stress Questionnaire – Family Stress Version)	80%
**Svanberg, Stott & Spector.** 'Just Helping': children living with a parent	2010	UK (no further information)	MM. (interview + survey)	12	6/9	Range=11-17<br>Mean=14.6	All parents, aged 39-59 years (Mean=50.7)	5 Alzheimer's disease<br>2 Pick's disease<br>2 vascular dementia<br>1 no formal diagnosis made, although Pick's	• Perceived normality of role and identifying as a carer (pride in their caring roles)<br>• Caring demands: Recent Mood and Feelings	60%

Supplementary Table S1. Summary of study characteristics of identified studies, including MMAT score

Source	Year	Setting	Design (QT/QL/MM)	YIC Sample Size	Male/ Female	Age, Mean (SD) or range	Relationship to care-recipient	Illness of care-recipient	Theme/measures used	MMAT Score (%)
with young onset dementia.								disease had been suspected	Questionnaire, Zarit Burden Interview-Short<br>• Coping strategies: Resilience Scale	
**Ireland & Pakenham.** Youth adjustment to parental illness or disability: The role of illness characteristics, caregiving, and attachment.	2010	Australia	QT (survey)	81	28/53	Range=10-25<br>Mean 16.31 (3.36)	56 Mothers<br>17 Fathers<br>8 Both	28 Physical illness/disability<br>35 Mental Illness<br>18 Both	• Social Support from formal and unpaid networks (young poepl who seek support better adjust to caring)<br>• Caring demands: Young Caregiver of Parents Inventory (YCOPI), Strengths and Difficulties Questionnaire (emotional symptoms, conduct problems, hyperactivity/inattention, peer problems and prosocial behaviour)<br>• Coping strategies: (resiliency – Inventory of Parent and Peer Attachment)	80%
**Moore, McArthur & Noble-Carr.** Different but the same? Exploring the experiences of young people caring for a parent with an	2011	Australia	QL (interview)	15	7/8	Range=11-17	8 Mothers<br>5 Fathers	Substance abuse problems	• Perceived normality of role and identifying as a carer ("doesn't sound right" especially if lower levels of care)<br>• Social Support from formal and unpaid networks (isolation and prosocial behaviour; need for	100%

Supplementary Table S1. Summary of study characteristics of identified studies, including MMAT score

Source	Year	Setting	Design (QT/QL/MM)	YIC Sample Size	Male/ Female	Age, Mean (SD) or range	Relationship to care-recipient	Illness of care-recipient	Theme/measures used	MMAT Score (%)
alcohol or other drug issue.									empathetic, responsive healthcare professionals)<br>• Caring demands (types of caring, impact of caring, stigma, isolation, needs of young people)<br>• Coping strategies (resiliency)<br>• Demographic factors: SES	
**Smyth, Blaxland & Cass.** 'So that's how I found out I was a young carer and that I actually had been a carer most of my life'. Identifying and supporting hidden young carers.	2011	Australia	QL (interview)	68	N/A<br><br>(mentions of gender through quotes)	Range=11 -25	Mentions of mothers and siblings	N/A<br><br>(mentions of cared-for siblings having mental disabilities and visual impairment)	• Perceived normality of role and identifying as a carer (disclosure of caring identity; recognising oneself as carer through interaction with others, caring embedded within a normative framework of familial obligations and responsibilities)<br>• Social Support from formal and unpaid networks (stigma compounded by low-income; bullying and home-schooling)	100%
**Barry.** 'I realised that I wasn't alone': The views and experiences of young carers from a social	2011	Scotland, UK	QL (interview)	20	10/10	Range=12 -23	9 Mothers<br>4 Siblings<br>4 Both parents<br>2 Father<br>1 Stepfather	N/A<br><br>(Mentions of mental or physical disabilities, mental health problems, ADHD, alcoholism and physical illnesses)	• Perceived normality of role and identifying as a carer (different perceptions – no resentment but tensions within household)<br>• Social Support from formal and unpaid networks	100%

Supplementary Table S1. Summary of study characteristics of identified studies, including MMAT score

Source	Year	Setting	Design (QT/QL/MM)	YIC Sample Size	Male/ Female	Age, Mean (SD) or range	Relationship to care-recipient	Illness of care-recipient	Theme/measures used	MMAT Score (%)
capital perspective									(teachers, schools, friends, worry and guilt)<br>• Caring demands (stigma/embarrassment, worry about the future, school as respite)<br>• Coping strategies (family closeness)	
**Moyson & Roeyers.** The overall quality of my life as a sibling is all right, but of course, it could always be better'. Quality of life of siblings of children with intellectual disability: the siblings' perspectives	2012	Belgium	QL (interview)	50	23/27	Range=6-14 Mean=9.16 (2.04)	All Siblings (30 Younger, 18 Older, 2 Twin)	Intellectual disability	• Social Support from formal and unpaid networks (respite, support from parents)<br>• Caring demands (stress from misunderstanding, respite)<br>• Coping strategies (ignoring behaviour, trying to understand, adapting to it, isolating themselves)	100%
**Graff, Mandleco, Dyches et al.** Perspectives of adolescent siblings of children with Down syndrome	2012	United States	QL (interview)	23	11/12	Range=12-19 Mean= 16 (2.15)	Younger siblings, mean age 12 years (SD = 2.53)	Down Syndrome	• Caring demands (negative aspects of caring, impact on family and parents)<br>• Coping strategies (positive aspects of caring)	100%

Supplementary Table S1. Summary of study characteristics of identified studies, including MMAT score

Source	Year	Setting	Design (QT/QL/MM)	YIC Sample Size	Male/ Female	Age, Mean (SD) or range	Relationship to care-recipient	Illness of care-recipient	Theme/measures used	MMAT Score (%)
who have multiple health problems										
**Hamilton & Adamson.** Bounded agency in young carers' life course-stage domains and transitions.	2013	Australia	QL (interview)	33	19/14	Range=7-25	16 Mothers<br>7 Fathers<br>18 Siblings + others	23 Physical illness<br>17 Mental Illness<br>13 Intellectual disability<br>12 Long-term illness<br>11 Sensory difficulties<br>6 AOD<br>11 imited mobility/other	• Perceived normality of role and identifying as a carer (gradual pathway into care, perceived constraints)<br>• Social Support from formal and unpaid networks (social relationships, services and gaps identified)<br>• Caring demands (health and wellbeing, questions and constraints over thinking of the future, employment, and education)	100%
**Collins & Bayless.** How caring for a parent affects the psychosocial development of the young.	2013	UK (no further information)	QT (survey)	20	N/A	Range=11-18	Parent	N/A	• Caring demands: Strengths and Difficulties Questionnaire (behavioural strengths and difficulties), Satisfaction with Life Scale (life satisfaction), Visual Analogue Self-Esteem Scales (self-esteem)	80%
**Heyman & Heyman.** 'The sooner you can change their life course the better':	2013	England, UK	QL (interview)	13	N/A<br><br>(mentions gender	Range=16-29	N/A	N/A	• Perceived normality of role and identifying as a carer (not seeing themselves as vulnerable)	60%

Supplementary Table S1. Summary of study characteristics of identified studies, including MMAT score

Source	Year	Setting	Design (QT/QL/MM)	YIC Sample Size	Male/ Female	Age, Mean (SD) or range	Relationship to care-recipient	Illness of care-recipient	Theme/measures used	MMAT Score (%)
the time-framing of risks in relationship to being a young carer					through quotes)				• Social Support from formal and unpaid networks (services not perceived as helpful to help them plan future, insensitive education system)<br>• Caring demands (identification of stresses)<br>• Coping strategies (identifications of positives)	
**Ali, Ahlström, Krevers et al.** Support for young unpaid carers of persons with mental illness: A mixed-method study	2013	Sweden	MM. (interview + survey)	253	74/179	Range=16 -15	N/A	Mental illness	• Social Support from formal and unpaid networks (isolation, meaningful relationships, very few had professional support, but some said web-based support was helpful)<br>• Caring demands (loneliness, anxiety)	80%
**Nichols, Fam, Cook et al.** When dementia is in the house: Needs assessment survey for young caregivers	2013	Canada	QL (interview)	14	4/10	Range=11 -18	Parent	Dementia (FTD)	• Perceived normality of role and identifying as a carer (balancing caring with youth)<br>• Social Support from formal and unpaid networks (friends; family; guidelines from health professionals)<br>• Caring demands (emotional impact)	100%

Supplementary Table S1. Summary of study characteristics of identified studies, including MMAT score

Source	Year	Setting	Design (QT/QL/MM)	YIC Sample Size	Male/ Female	Age, Mean (SD) or range	Relationship to care-recipient	Illness of care-recipient	Theme/measures used	MMAT Score (%)
									• Coping strategies (sense of reward, relationship with family)	
**Sieh, Visser-Meily, & Meijer.** Differential Outcomes of Adolescents with Chronically Ill and Healthy Parents	2013	Netherlands	QT (survey)	161	77.924/ 83.08	Range=10 -20 Mean=15. 1 (2.3)	Parent (67% female)	Multiple sclerosis (28.2%)<br>Rheumatoid arthritis (19.4%)<br>Brain damage (16.5%)<br>Muscle disease (14.6%)<br>Spinal cord injury (6.8%)<br>Inflammatory bowel disease (5.8%)<br>Parkinson disease (5.8%)<br>Diabetes type I with physical complications (2.9%)	• Caring demands (Young Caregiver of Parents Inventory (YCOPI), Daily Hassles Questionnaire (Stress and Coping Variables), Youth self-report of problem behaviour)<br>• Social Support from formal and unpaid networks (Inventory of Parent and Peer Attachment; isolation and prosocial behaviour)<br>• Coping strategies (Daily Hassles Questionnaire)	80%
**Llyod.** Happiness and Well-Being of Young Carers: Extent, Nature and Correlates of Caring Among 10 and 11 Year Old School Children	2013	Northern Ireland, UK	QT (survey)	192/419 2	Of total sample: 1970.24/22 21.76	Range=10 -11	Grandmother (44%),<br>Mother (33%)<br>Brother (33%)<br>Grandfather (27%)<br>Father (26%)<br>Sister (26%)<br><br>57% of the children helped look after one person<br>16% helped look after two people	N/A	• Caring demands: KIDSCREEN (health-related quality of life)	60%

Supplementary Table S1. Summary of study characteristics of identified studies, including MMAT score

Source	Year	Setting	Design (QT/QL/MM)	YIC Sample Size	Male/ Female	Age, Mean (SD) or range	Relationship to care-recipient	Illness of care-recipient	Theme/measures used	MMAT Score (%)
							27% helped to look after three or more people			
**Cassidy, Giles, & McLaughlin.** Benefit finding and resilience in child caregivers	2014	UK (no further information)	QT (survey)	442	174/268	Range=12-16<br>Mean=13.4 (1.38)	203 Parent<br>168 Sibling<br>71 More than one family member	143 chronic illness<br>106 learning disability<br>47 physical disability<br>76 mental illness<br>70 with a combination of caring needs	• Social Support from formal and unpaid networks: Perceived Social Support Scale<br>• Caring demands: The General Health Questionnaire, The Perceived Impact of Child Caregiving Scale<br>• Coping strategies: Adolescent Coping Scale-short form, The Brief Resilience Scale, The Benefit-Finding in Child Caregivers Scale	60%
**Millenaar, Van Vliet, Bakker et al.** The experiences and needs of children living with a	2014	Netherlands	QL (interview)	14	6/8	Range=15-27<br>Mean=21	Parent<br>3 Mothers<br>11 Fathers<br><br>The mean age of the parent was 53.6	5 Alzheimer's disease<br>4 fronto-temporal dementia (2 with the behavioral variant and 2 with primary progressive aphasia)	• Social Support from formal and unpaid networks (children need of care and support)	100%

Supplementary Table S1. Summary of study characteristics of identified studies, including MMAT score

Source	Year	Setting	Design (QT/QL/MM)	YIC Sample Size	Male/ Female	Age, Mean (SD) or range	Relationship to care-recipient	Illness of care-recipient	Theme/measures used	MMAT Score (%)
parent with young onset dementia: Results from the NeedYD study							years (SD 4.5, range 47–62)	1 vascular dementia<br>1 dementia not otherwise specified	• Caring demands (impact of dementia on daily life, anxieties about future)<br>• Coping strategies (different ways of coping with the disease)	
**Pakenham, & Cox.** Comparisons between youth of a parent with MS and a control group on adjustment, caregiving, attachment and family functioning.	2014	Australia	QT (survey)	126	58/68	Mean=14.04 (3.00)	Parent	Multiple Sclerosis	• Social Support from formal and unpaid networks: Inventory of Parent and Peer Attachment, Family Environment Scale<br>• Caring demands: Young Carers of Parents Inventory (YCOPI), Strengths and Difficulties Questionnaire, Brief Symptom Inventory	80%
**Kavanaugh.** Children and Adolescents Providing Care to a Parent with Huntington's Disease: Disease Symptoms, Caregiving Tasks and Young Carer Well-Being	2014	United States	MM (interview + survey)	40	9/31	Range=12-20	40 Parents<br>25 Mothers<br>15 Fathers	Huntington's Disease	• Caring demands: Young Carers of Parents Inventory (YCOPI), Children's Depression Inventory, Conflict Behaviour Questionnaire, the Multidimensional Assessment of Caring Activities (MACA)	60%

Supplementary Table S1. Summary of study characteristics of identified studies, including MMAT score

Source	Year	Setting	Design (QT/QL/MM)	YIC Sample Size	Male/ Female	Age, Mean (SD) or range	Relationship to care-recipient	Illness of care-recipient	Theme/measures used	MMAT Score (%)
**Pakenham & Cox.** The effects of parental illness and other ill family members on youth caregiving experiences.	2015	Australia	QT (survey)	706/2474	967.04/1506.96 (of total sample)	Range=9-20	336 Parents<br>254 Other, including:<br>96 Grandparents<br>99 Siblings<br>116 Both	Physical illness or disability, Mental illness, Alcohol and/or drug problems	• Caring demands: Young Carers of Parents Inventory (YCOPI)	100%
**Kavanaugh, Noh & Studer.** "It'd be nice if someone asked me how I was doing. Like, 'cause I will have an answer": exploring support needs of young carers of a parent with Huntington's disease	2015	United States	QL (interview)	40	9/31	Range=12-20	40 Parents<br>25 Mothers<br>15 Fathers	Huntington's Disease	• Social Support from formal and unpaid networks (types of support: instrumental, emotional, and personal needs; peer support groups; isolation; difficulties in development of friendships)	100%
**Kavanaugh, Noh, Zhang et al.** Caregiving Youth Knowledge and Perceptions of Parental End-of-Life Wishes in	2016	United States	QL (interview)	40	9/31	Range=12-20	40 Parents<br>25 Mothers<br>15 Fathers	Huntington's Disease	• Perceived normality of role and identifying as a carer (caregiving responsibility)<br>• Social Support from formal and unpaid networks (respect of parental and caregiver wishes about the future and care)	100%

Supplementary Table S1. Summary of study characteristics of identified studies, including MMAT score

Source	Year	Setting	Design (QT/QL/MM)	YIC Sample Size	Male/ Female	Age, Mean (SD) or range	Relationship to care-recipient	Illness of care-recipient	Theme/measures used	MMAT Score (%)
Huntington's Disease										
**Meltzer.** 'I couldn't just entirely be her sister': The relational and social policy implications of care between young adult siblings with and without disabilities.	2017	Australia	QL (interview)	21/46	22/24 (of total sample)	Range=15 -29	25 Siblings	Disability	• Perceived normality of role and identifying as a carer (framing of the care, double identity – sister and carer)<br>• Social Support from formal and unpaid networks (helping within family)<br>• Caring demands (caring tasks)	100%
**Greene, Cohen, Siskowski et al.** The Relationship Between Family Caregiving and the Mental Health of Emerging Young Adult Caregivers.	2017	United States	QT (survey)	157/353 (non-carer comparison group)	34/123<br><br>(excludes non-carer: 36/160)	No Range. Mean=20. 79 (1.64)	Parent & Grandparent	N/A	• Caring demands: Center for Epidemiological Studies of Depression Scale, State-Trait Anxiety Inventory, Rosenberg Self-Esteem Scale, Response to Stress Questionnaire	100%
**Moberg, Larsen, & Brødsgaard.** Striving for balance between caring and	2017	Denmark	QL (interview)	14	2/12	Range=18 -25 Mean=20. 6	15 Parents<br>10 Mother<br>5 Father	Multiple Sclerosis	• Perceived normality of role and identifying as a carer (type of caring task and how they help identify as a carer)	100%

Supplementary Table S1. Summary of study characteristics of identified studies, including MMAT score

Source	Year	Setting	Design (QT/QL/MM)	YIC Sample Size	Male/ Female	Age, Mean (SD) or range	Relationship to care-recipient	Illness of care-recipient	Theme/measures used	MMAT Score (%)
restraint: young adults' experiences with parental multiple sclerosis									• Social Support from formal and unpaid networks (lack of openness and knowledge)<br>• Caring demands (participant emotions in reaction to caring, worry and guilt, shame, anxiety, depression)<br>• Coping strategies (choosing health professions, advantages of responsibility)	
**Lakman, Chalmers, & Sexton.** Young carers' educational experiences and support: A roadmap for the development of school policies to foster their academic success.	2017	Canada	QT (survey)	145	58/82 (5 undisclosed)	Range=8-18 Mean=12	N/A	N/A	Not mentioned – related to social support (education)	100%
**Bowman, Alvarez-Jimenez, Wade et al.** The positive and negative experiences of caregiving for siblings of young	2017	Australia	QT (survey)	157	81/76	Mean=21.7 (4.4)	157 Siblings<br>81 Brother<br>76 Sister	Early Psychosis	• Caring demands: Experience of Caregiving Inventory (mental health issues)<br>• Coping strategies: Experience of Caregiving Inventory	80%

Supplementary Table S1. Summary of study characteristics of identified studies, including MMAT score

Source	Year	Setting	Design (QT/QL/MM)	YIC Sample Size	Male/ Female	Age, Mean (SD) or range	Relationship to care-recipient	Illness of care-recipient	Theme/measures used	MMAT Score (%)
people with first episode psychosis.										
**Heyman.** What do young adult carers learn through supporting family members? Extending the affirmation model	2018	England, UK	QL (biographical accounts)	13	8/5	Range=16-30	N/A	Physical disability, Acute illness, Learning disability	• Caring demands (skills learned)<br>• Coping strategies (learning valued, personal growth, self-assertion)	100%
**Spratt, McGibbon, & Davidson.** Using Adverse Childhood Experience Scores to Better Understand the Needs of Young Carers.	2018	Northern Ireland, UK	MM (Interview + survey)	16/22	4/18	Range=8-18	Parent	Mental Illness	• Perceived normality of role and identifying as a carer (feeling used to caring due to caring for a long time)<br>• Caring demands: Adverse Childhood Experiences Questionnaire (mental illness, etc.)	60%
**Nuttall, Coberly, & Diesel.** Childhood Caregiving Roles, Perceptions of Benefits, and	2018	United States	QT (survey)	~68/108	N/A	Range=18-25<br>Mean=18.14 (5.49)	Siblings	Autism Spectrum Disorder<br>Autism<br>Asperger's<br>Pervasive Developmental Disability	• Perceived normality of role and identifying as a carer (Parentification Inventory, Intention for Caring Involvement in the Future)	80%

Supplementary Table S1. Summary of study characteristics of identified studies, including MMAT score

Source	Year	Setting	Design (QT/QL/MM)	YIC Sample Size	Male/ Female	Age, Mean (SD) or range	Relationship to care-recipient	Illness of care-recipient	Theme/measures used	MMAT Score (%)
Future Caregiving Intentions Among Typically Developing Adult Siblings of Individuals with Autism Spectrum Disorder.									• Coping Strategies: Autism benefit-finding scale	
**Leu, Frech, & Jung.** Young carers and young adult carers in Switzerland: Caring roles, ways into care and the meaning of communication.	2018	Switzerland	QL (interview)	29	5/23	Range=10 -25	19 Parents<br>16 Mothers<br>2 Fathers<br><br>9 siblings<br>1 Grandparent	15 Physical impairment<br>10 Mental Impairment<br>1 Addition<br>3 No diagnosis	• Perceived normality of role and identifying as a carer (pathways into caring; awareness of roles)<br>• Social Support from formal and unpaid networks (communication about the caring situation with family, professionals, friends, extended family and stigma)<br>• Caring demands (nature of caring roles and intensity of caring roles)	80%
**Boumans & Dorant.** A cross-sectional study on experiences of young adult carers compared to young adult noncarers:	2018	Netherlands	QT (survey)	297<br><br>School 1: 181<br>School 2: 116	65/232	Range=18 -24<br><br>School 1: Mean=18.9	N/A	Chronic disease, physical and intellectual disability	• Perceived normality of role and identifying as a carer (Parentification, Maastricht Parentification Scale)<br>• Coping Strategies: Brief Resilience Scale, Utrechtse Coping Lijst	100%

Supplementary Table S1. Summary of study characteristics of identified studies, including MMAT score

Source	Year	Setting	Design (QT/QL/MM)	YIC Sample Size	Male/ Female	Age, Mean (SD) or range	Relationship to care-recipient	Illness of care-recipient	Theme/measures used	MMAT Score (%)
parentification, coping and resilience						School 2: Mean=19.0				
**McDougall, O'Connor, & Howell.** "Something that happens at home and stays at home": An exploration of the lived experience of young carers in Western Australia.	2018	Australia	QL (interview)	13<br><br>1ary (n=4), 2ary carer (n=9)	6/7	Range=14-25 Mean=18	7 cared for parents and grandparents<br>7 cared for siblings<br>1 cared for cousins<br>1 cared for non-family member	Disability, mental illness, chronic condition, terminal illness, alcohol or other drug issue, or frail aged	• Perceived normality of role and identifying as a carer (not disclosing identity due to desire for normalcy and privacy)<br>• Social Support from formal and unpaid networks (solace in socialising and solidarity, lost in the system)<br>• Caring demands (navigating competing demands)<br>• Coping Strategies (Lessons from Experience, solace in solitude)	100%
**Kallander, Weimand, Becker et al.** Children with ill parents: extent and nature of caring activities	2018	Norway	QT (survey)	246	106.03/139.97	Range=8-17	238 Parents	135 physical illness (neurological disease or cancer)<br>75 mental illness<br>28 substance abuse	• Perceived normality of role and identifying as a carer: Family Adaptability and Cohesion Evaluation Scale<br>• Social Support from formal and unpaid networks: Interpersonal Support Evaluation List<br>• Caring demands: Multidimensional Assessment of Caring Activities (MACA), Social	100%

Supplementary Table S1. Summary of study characteristics of identified studies, including MMAT score

**Source**	**Year**	**Setting**	**Design (QT/QL/MM)**	**YIC Sample Size**	**Male/ Female**	**Age, Mean (SD) or range**	**Relationship to care-recipient**	**Illness of care-recipient**	**Theme/measures used**	**MMAT Score (%)**
									Skills Rating System, Health Survey SF-8, Hopkins Symptoms Checklist, Family Adaptability and Cohesion Evaluation Scale<br>• Coping Strategies: Family Adaptability and Cohesion Evaluation Scale	
**Stamatopoulos.** The young carer penalty: Exploring the costs of caregiving among a sample of Canadian youth	2018	Canada	QL (interview + focus groups)	15	3/12	Range=15 -19<br>Mean=16	Siblings (50%+)<br>Parent<br>Both	Substance Abuse (alcohol)<br>Terminal Cancer<br>Autism<br>Comorbid conditions<br>Physical illness and/or disability (e.g., late-stage cancer, stroke, and diabetes resulting in double amputation).	• Perceived normality of role and identifying as a carer (balancing caring with youth; disclosure of caring identity)<br>• Social Support from formal and unpaid networks (friendship sustainability and social media use; isolation; future decisions with care-recipient in mind)<br>• Caring demands: personal penalties like familial strain, limited social opportunities, emotional health and wellbeing, penalties in education and employment<br>• Coping Strategies: benefits of youth-based caregiving	100%

Supplementary Table S1. Summary of study characteristics of identified studies, including MMAT score

Source	Year	Setting	Design (QT/QL/MM)	YIC Sample Size	Male/ Female	Age, Mean (SD) or range	Relationship to care-recipient	Illness of care-recipient	Theme/measures used	MMAT Score (%)
**Becker & Sempik.** Young Adult Carers: The Impact of Caring on Health and Education.	2019	UK (no further information)	QT (survey)	295	62/231	Range=14-25<br>Mean=18.8	154 mother<br>41 father<br>12 both parents<br>63 cared for siblings/step-siblings<br>12 cared for grandparents<br><br>200 (68%) cared for 1 person, 72 (24%) cared for two people, 10 (3%) cared for three people, 13 (4%) cared for four+ people	163 Physical disability<br>139 Long term illness<br>144 Mental ill health<br>69 Learning disabilities<br>34 Old Age<br>13 Dementia	• Caring demands: Multidimensional Assessment of Caring Activities (MACA) for level of caring and perceived health, perceptions of school, college and university, impact of caring on education	80%
**Järkestig-Berggren, Bergman, Eriksson, et al.** Young carers in Sweden—A pilot study of care activities, view of caring, and psychological well-being	2019	Sweden	MM (survey + focus groups	30	7/23	Range=10-18	21 Mother<br>19 Sibling<br>15 Father<br>16 children explicitly stated that they help more than one person.	Parental addiction, mental health problems, or violence against a parent	• Social Support from formal and unpaid networks (perceptions of caring based on family recognition and support)<br>• Caring demands: Multidimensional Assessment of Caring Activities (MACA) and Positive and Negative Outcomes of Caring (PANOC), KIDSCREEN and Strengths and Difficulties Questionnaire for health-	80%

Supplementary Table S1. Summary of study characteristics of identified studies, including MMAT score

Source	Year	Setting	Design (QT/QL/MM)	YIC Sample Size	Male/ Female	Age, Mean (SD) or range	Relationship to care-recipient	Illness of care-recipient	Theme/measures used	MMAT Score (%)
									related quality of life and psychological wellbeing<br>• Demographic variables: Family Affluence Scale	
**McGibbon, Spratt, & Davidson.** Young Carers in Northern Ireland: Perceptions of and Responses to Illness and Disability within the Family.	2019	Northern Ireland, UK	QL (interview)	22	4/18	Range=8-18<br>Mean=14	Parent and sibling<br>11 young carers were providing care for more than one person	16 mental health problems<br>8 comorbid physical/mental illness)<br>Care-recipients under 18: cerebral palsy, ADHD and autism.	• Perceived normality of role and identifying as a carer (choice in caregiving, feelings of obligation, awareness of role through interaction with other carers)<br>• Caring demands (impact of physical illness, spreading out responsibility and caring for sibling)<br>• Coping Strategies (heightened intuition, building resilience, gratitude and closeness with sibling)	100%
**Lakman, & Chalmers.** Psychosocial comparison of carers and noncarers.	2019	Canada	QT (Comparative Analysis)	124/248	52/72	Range=10-17<br>(Mean unclear)	N/A	N/A	• Caring demands: Dimensions of Temperament Survey, Youth Leisure Study and Life Optimism Test, Depression Scale (CES-D), Rosenberg's self-esteem scale, Ginsburg, LaGreva and Silverman social Anxiety scale, Louvain Loneliness Scale for Children and Adolescence	80%

Supplementary Table S1. Summary of study characteristics of identified studies, including MMAT score

Source	Year	Setting	Design (QT/QL/MM)	YIC Sample Size	Male/ Female	Age, Mean (SD) or range	Relationship to care-recipient	Illness of care-recipient	Theme/measures used	MMAT Score (%)
									• Social Support from formal and unpaid networks: Louvain Loneliness Scale for Children and Adolescence & Ginsburg, LaGreva and Silverman social Anxiety scale	
